# Dating Sediments by EPR Using Al-h Centre: A Comparison between the Properties of Fine (4–11 µm) and Coarse (>63 µm) Quartz Grains

**DOI:** 10.3390/molecules27092683

**Published:** 2022-04-21

**Authors:** Zuzanna Kabacińska, Alida Timar-Gabor

**Affiliations:** 1 Interdisciplinary Research Institute on Bio-Nano-Sciences, Babeș-Bolyai University, Treboniu Laurian 42, 400271 Cluj-Napoca, Romania; alida.timar@ubbcluj.ro; 2 Faculty of Environmental Science and Engineering, Babeș-Bolyai University, Fântânele 30, 400000 Cluj-Napoca, Romania

**Keywords:** electron paramagnetic resonance (EPR), electron spin resonance (ESR), quartz, Al-h centre, fine grains, dose response curve

## Abstract

The possibility of EPR dating for sediments using Al-h signals of fine (4–11 μm) grains of quartz has not been previously discussed. Here, the Al-h and peroxy EPR spectra of fine (4–11 μm) and coarse (63–90, 125–180 μm) sedimentary quartz from thoroughly investigated loess sites in Eastern Europe were examined. By comparing experimental spectra with a simulated signal, we evaluated the overestimation observed when using the standard approach established by Toyoda and Falguères to measure Al-h intensity for different doses of radiation, up to 40,000 Gy. This overestimation, caused by the presence of peroxy signals, was much more pronounced for fine grains. Fine grains exhibited some additional dose-dependent signals, which, for some samples, caused a complete distortion of the Al-h spectra at high doses, making it impossible to measure the standard amplitude. We propose a new approach to measuring Al-h signal intensity, focusing on the peak-to-baseline amplitude of the part of the signal at g ≈ 2.0603, which is not affected by the peroxy signals and therefore has the potential of providing more accurate results. The shapes of dose response curves constructed for coarse and fine grains using the new approach show considerable similarity, suggesting that Al-h centre formation in fine and coarse grains upon artificial radiation at room temperature follows the same pattern.

## 1. Introduction

Quartz (SiO_2_) is a material of great importance in many areas of Earth sciences, as well as in industry. As all crystals, it contains a vast number of point defects, which may be either intrinsic (involving only atoms of the host lattice—vacancies, interstitial atoms and excess atoms) or extrinsic (belonging to foreign atoms in lattice and inter-lattice positions) [1,2]. Those of most interest in the field of geochronology include Si- and O-vacancies and impurity related defects. Among the latter, Al^3+^ always presents in quartz, substituting for Si^4+^ with charge compensation generally achieved by Li^+^, Na^+^ or H^+^, which gives rise to [AlO_4/_M^+^]^0^ (where M^+^ denotes an alkali metal or hydrogen ion) [3]. Ti^4+^ may substitute for Si^4+^ in quartz with no charge compensation, creating [TiO_4_]^0^ [4]. Ge centre, namely [GeO_4_/M^+^]^0^ (most notably [GeO_4_/Li^+^]^0^) is sometimes observed in irradiated natural quartz [4,5]. A neutral oxygen vacancy can trap an electronic hole, forming a paramagnetic oxygen vacancy (E_1_′ centre) [4,5]. Performing systematic investigations on quartz using electron paramagnetic resonance (EPR) (or electron spin resonance—ESR) spectroscopy, a method of high sensitivity, allows for gaining a deeper understanding of the mechanisms involved when the defects in quartz are subjected to irradiation.

EPR has been applied in dating geological and archaeological materials for over 40 years. Together with optically stimulated luminescence (OSL) and thermoluminescence (TL), the so-called trapped-charge dating methods have been extensively used for dating sediments using quartz (e.g., [6,7,8,9]). Quartz records the amount of ionising radiation it has been exposed to as a latent signal within its crystal lattice, and therefore can be used as a natural dosimeter for quantifying the radiation history of materials. Irradiation at room temperature leads to the dissociation of [AlO_4/_M^+^]^0^, resulting in the formation of [AlO_4/_H^+^]^0^ and [AlO_4_]^0^ [10]. [AlO_4_]^0^, also referred to as Al-hole or Al-h, as a paramagnetic centre, is therefore detectable by EPR, and has been extensively used for dating sediments [6,7,9,11,12,13,14,15,16,17,18,19]. Ti centres have also been widely used for dating [6,11,18,19,20]. Upon room temperature irradiation, Ti^4+^ may trap an electron together with an alkali ion M^+^ for charge compensation, forming [TiO_4_/M^+^]^0^, where M^+^ can be either Li^+^, H^+^ or Na^+^ [4]. Trapped-charge dating methods are based on the assumption that the natural growth of the signal of interest can be reproduced by laboratory irradiation, which leads to the construction of a dose response curve—a plot of EPR intensity versus the doses of irradiation, obtained separately for every investigated sample. The equivalent dose—a total dose of radiation absorbed by the crystal, giving rise to the signal measured in the natural sample—is determined by extrapolation (in the case of additive dose protocols) or interpolation (in the case of regenerative protocols) of the dose response curve.

In many luminescence and EPR dating studies, the choice of grain size fraction used for analysis has been most often dictated predominantly by the nature and availability of the material. Based on a series of previous research, Timar-Gabor et al. [21] showed that there is a discrepancy in the ages obtained by the single aliquot regeneration protocol (SAR) OSL between different grain sizes and an age underestimation for finer grains, and suggested a potentially worldwide phenomenon. However, defects giving rise to luminescence in quartz have not yet been unambiguously identified, and their correlation with the defects detected by EPR remains unestablished. Consequently, observations concerning grain size effects based on luminesce results are not directly transferable to EPR defects, which leaves this topic largely unexplored.

To fill this gap, a systematic approach needs to be employed, starting with a thorough investigation of the dependence of EPR intensity of defects on grain size, and followed by experiments showing their behaviour when subjected to laboratory irradiation, which would expose any possible differences between fine- and coarse-grained quartz. It goes without saying that such experimental studies should also be complemented by the development of appropriate models. The first objective was addressed by Timar-Gabor [22] by showing the dependence of EPR intensity of the main paramagnetic defects in quartz with grain size, for fraction 4–11, 63–90, 90–125, 125–180, and 180–250 μm. The intensity of the E_1_′ and Al-h signal in natural samples was found to decrease with increasing grain size, while [TiO_4_/Li^+^]^0^ signals, detected only in coarse fractions, increased with increasing grain size. The second objective, the investigation the behaviour of the defects under laboratory irradiation, is the subject of this work. To achieve any of these goals, or, in fact, to obtain any reliable dating, an accurate measurement of EPR signal intensity is crucial.

In this study, we focus on the Al-h signal in fine (4–11 μm) and coarse (>60 μm) grains. The Al-h signal can only be measured by EPR at cryogenic temperatures due to the very short spin–lattice relaxation time of the defect. It produces a complex EPR spectrum arising from the interaction of the unpaired electron with nearby magnetic nuclei. The Al-h signal consists of a central set of peaks around g ≈ 2.008 displaying a distinct hyperfine structure, and a much less intense set of peaks at about g ≈ 2.06. In early attempts at dating using the Al-h signal [23,24,25], different peaks from the central set were considered for evaluating its intensity. Their reliability was compared in a study by Lin et al. [26]. Eventually, a common approach proposed by Toyoda and Falguères [27] was adopted, and it has been widely used ever since by the EPR dating community (e.g., [6,9,15,16,17]). This approach is based on the measurement of a peak-to-peak amplitude between the top of the first peak (g = 2.018) and the bottom of the last peak (g = 1.993) of the central part of the ^27^Al hyperfine structure. This method has been extremely useful due to its simplicity and the fact that it focuses on the most distinct peaks, which are clearly distinguishable even for very weak signals. However, its applicability is sometimes limited by the presence of additional signals superimposed on the central part of the Al-h signal.

These additional signals are referred to as “peroxy” species or, for simplicity, sometimes as a peroxy centre (singular), although their spectrum is clearly composed of many overlapping signals. They are visible most clearly at room temperature, when the Al-h is not detectable. Friebele et al. [28] first established a peroxy radical in neutron or gamma-ray irradiated ^17^O-enriched fused silica and suggested it derives from pre-existing bridging peroxy linkages (≡Si–O–O–Si≡, where ≡ represents three Si-O bonds), which shed an electron to form peroxy radicals by irradiation and/or thermal treatment. Peroxy radicals in crystalline SiO_2_ (α-quartz) have been suggested by several EPR studies and established by Botis et al. [29,30], Nilges et al. [31,32] and Pan et al. [33,34] in their very detailed studies. Based on their research, it was concluded that most of the discrepancies in the literature concerning the g-factor values, linewidths and hyperfine structure reported for the peroxy centres can be attributed to incompletely resolved site splittings in previous X- and Q-band studies. For an in-depth investigation of these species, higher microwave frequencies should be applied, but the accessibility of such equipment is very limited. Despite the wealth of information provided by these studies, there are still many unanswered questions regarding the nature of these signals, answers to which are essential considering their relevance in EPR dating and provenance investigations. It should be noted that, apart from peroxy centres, another type of oxygen excess centre has been identified, namely, the non-bridging oxygen hole centre (NBOHC), described as oxygen dangling bonds ≡Si–O∙ (where ∙ represents an unpaired electron) [35]. For simplicity, however, in this study, we use the term “peroxy” to describe all signals observed between g ≈ 2.01 and g ≈ 1.99 at room temperature, with the exception of E’ and Ge centres.

The complexity of the peroxy spectrum, combined with the limitations of the X-band spectroscopy routinely used for dating, makes attempts at isolating these signals to obtain an undisturbed signal from the Al-h centre extremely challenging. Perhaps for this reason, the issue of peroxy signals interfering with Al-h measurements has been largely ignored in the literature. However, some amendments have been occasionally employed to circumvent the problem, such as subtracting the overlapping peroxy signal intensity using its EPR signal intensity after annealing [36]. The assumption here is that the peroxy signal changes neither with heating nor with the dose of irradiation, and the same signal can be used for subtraction at low and high doses. While the latter assumption is generally accepted in the case of coarse grains, this has not been confirmed for fine grains. Indeed, Timar-Gabor [22] reported on a dose-dependent signal at g ≈ 2.011, detected in a fine-grained fraction of quartz, which suggests that this approach might not be applicable in every case. Moreover, it introduces additional uncertainty related to the determination of peroxy signal intensity. Another approach, used by Tsukamoto et al. [7] for Al-h measurements conducted at 123 K, when the ^27^Al hyperfine structures were not visible, was based on the measurement of the peak-to-peak intensity of the first central peak. It was reported to be consistent with the peak-to-peak intensity of the whole peak minus the intensity of the peroxy centre, which was measured at 183 K. No significant changes in the peroxy centre intensity were observed when raising the temperature from 123 to 183 K, and the Al-h signal at 183 K became almost undetectable. As in the previous example, this method bears some additional uncertainties. Additionally, the authors noted that their measurements might not be directly comparable with other studies, which use the traditional approach. It is clear that developing an alternative approach to measuring Al-h signal intensity, unaffected by the presence of the peroxy signals, would improve the accuracy of age determination and, therefore, greatly benefit the EPR dating community.

The aforementioned study by Timar-Gabor [22], conducted on several samples, including two (ROX 1.14 and STY 1.10) studied in this work, shows that peroxy signals have significantly higher intensities in fine grains (4–11 μm) and decrease when grain size increases. Extended etching experiments resulted in obtaining partial evidence that these defects are concentrated in damaged areas of the grains. The weaker signals of peroxy centres would suggest that coarser fractions should be preferred for conventional EPR dating using the approach of Toyoda and Falguères [27] to measure Al-h intensity. However, assuming that the issue of accurately determining Al-h signal intensity could be solved, finer grains would not have to be automatically dismissed solely on that basis, especially as they are the main constituents of many sedimentary archives, such as loess, lake or marine sediments. That would allow for a thorough comparison of the properties of the Al-h signal observed in fine and coarse grains and open the possibility of EPR dating based on fine grains, which has not been discussed before.

In this work, we propose an alternative method of evaluating Al-h signal intensity, which circumvents the issue of interfering peroxy signals. The results obtained for the new approach and the standard approach by Toyoda and Falguères [27] are compared using the measurements of fine (4–11 μm) and coarse (63–90, 125–180 μm) quartz separates from thoroughly investigated loess palaeosol sites in Eastern Europe (Roxolany, Stayky and Mircea Vodă), which were used in the previous investigations carried out by our group. By comparing the experimental spectra with a simulated signal of the Al-h centre, we evaluated the overestimation that results from using the standard approach for different doses of radiation, up to 40,000 Gy. We then used the dose response curves constructed from the intensities obtained with the new approach to compare, for the first time, the response of the Al-h signal to laboratory irradiation displayed by fine- and coarse-grained fractions.

## 2. Materials and Methods

### 2.1. Samples

Experiments presented in this study were conducted on archived quartz separates of different grain sizes from previous investigations carried out by our group. 

Sample Rox 1.14 originates from Roxolany, loess palaeosol section, Southern Ukraine, and was collected below the Brunhes/Matuyama polarity transition. The results of EPR dating using a multicentre approach, along with optically stimulated investigations using both the standard single aliquot regenerative (SAR) multigrain OSL procedure, as well as single grain investigations, are presented in detail in [37]. 

Quartz sample Sty 1.10 comes from Stayky, loess palaeosol section, Northern Ukraine. The OSL chronology of this section, as well as extended SAR-OSL dose response curves on the Styky samples, are presented in detail in [38].

Sample 2 MV 80 was collected near the village of Mircea Vodă, which is situated in the Dobrogea plateau of SE Romania, about 15 km from the Danube River. Optical dating results for this site were published in [39] (including this sample) and in [8,40] (previous sampling).

Preparation protocol, following the standard OSL preparation guidelines, is described in detail in the aforementioned references, as well as in [22]. 

The selection of samples for the current study was based on the availability of sufficient material for EPR investigation and the high purity of the quartz extracts, as confirmed by routine tests in OSL dating as well as by scanning electron microscopy imaging, coupled with energy dispersive X-ray spectroscopy (EDX) [22].

### 2.2. EPR Measurements

EPR measurements were performed on an X-band Bruker EMX Plus spectrometer at Babeș-Bolyai University, Cluj-Napoca, Romania. All samples were placed in quartz glass tubes filled with a mass of 200 mg ± 10% for coarse grains (>63 μm) and 100 mg ± 10% for fine grains, maintaining the same volume, and with measurements later normalized to 100 mg for intercomparison. Care was taken that all samples were centred inside the cavity. Samples were rotated in the cavity using a programmable goniometer and measured at 3 different angles (every 120°, 1 scan per angle). Measurements were usually repeated 2–5 times at a few weeks’ intervals. Details of reproducibility tests are described in [22]. A mean EPR intensity was used for constructing a dose response curve, and standard error was indicated in all the plots. Exposure of samples to sunlight during measurements was restricted to a minimum. Measurements were carried out at 90 K for Al-h centres and at room temperature (295 K) for peroxy centres, using a variable temperature unit. Spectra were acquired using the following settings: 3350 ± 150 G scanned magnetic field, modulation amplitude 1 G, modulation frequency 100 kHz, microwave power 2 mW, conversion time 40 ms, time constant 40 ms. Baseline correction was performed when necessary using Bruker’s Xenon software.

Samples were gamma-irradiated with doses up to 10,000 Gy on top of the natural dose for sample ROX 1.14, and up to 40,000 Gy for samples STY 1.10 and 2 MV 80. Due to the limited availability of the material, fewer aliquots were obtained for the coarse fraction than for the fine fraction. Gamma irradiations were performed at room temperature at the Department of Health Technology at DTU (Dosimetry Research Unit) in Denmark using a calibrated ^60^Co gamma cell with a dose rate of 2 Gy/s (dose rate to water) at the time of irradiation. Dose rate to quartz was estimated to be 96% of dose rate to water based on Monte Carlo simulation considering the irradiation geometry used, as in [22].

### 2.3. Al-h Signal Simulations

**Figure 1:**
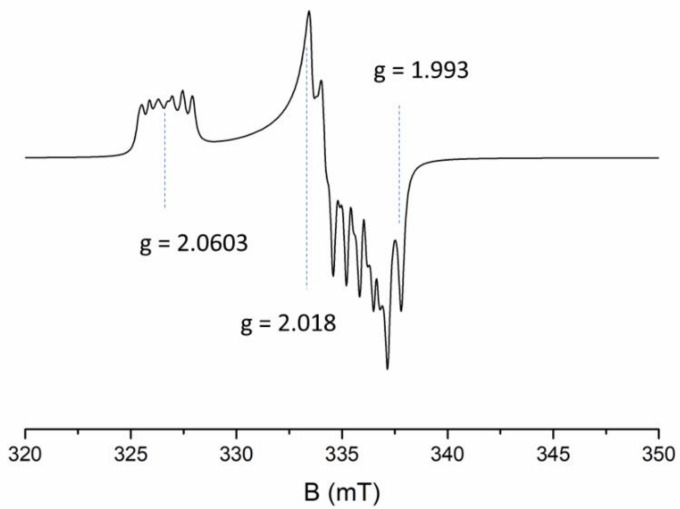
A simulated EPR signal of the Al-h centre with the g-factor values mentioned in the text. The parameters used for simulation are listed in Table 1.

Al-h signal was simulated with EasySpin [41] using parameters listed in Table 1. Initial parameters were based on [5,42] and adjusted to fit the experimental spectra. The values of quadrupole splitting were used as in [42] with no adjustments. When comparing with an experimental spectrum, an average of baseline-corrected experimental spectra obtained for a given dose was used. A spectrum recorded at a microwave frequency of 9.42 GHz was chosen as a reference, and magnetic field values for all spectra were adjusted to match the position of the signal. A simulated spectrum is shown in Figure 1, together with the principal components of the g-tensor values mentioned in the text.

## 3. Results and Discussion

### 3.1. Contribution of Peroxy Signals to Al-h Signal Measurements

The spectra of the Al-h and peroxy signals obtained for the coarse and fine grains of the quartz irradiated with different doses were compared based on three examples: sample ROX 1.14, STY 1.10 and 2 MV 80. Due to the different sources of quartz, one would expect these three samples to have different types and concentrations of defects, which makes them great subjects for studying the diversity of signals recorded by EPR.

#### 3.1.1. Sample ROX 1.14

**Figure 2:**
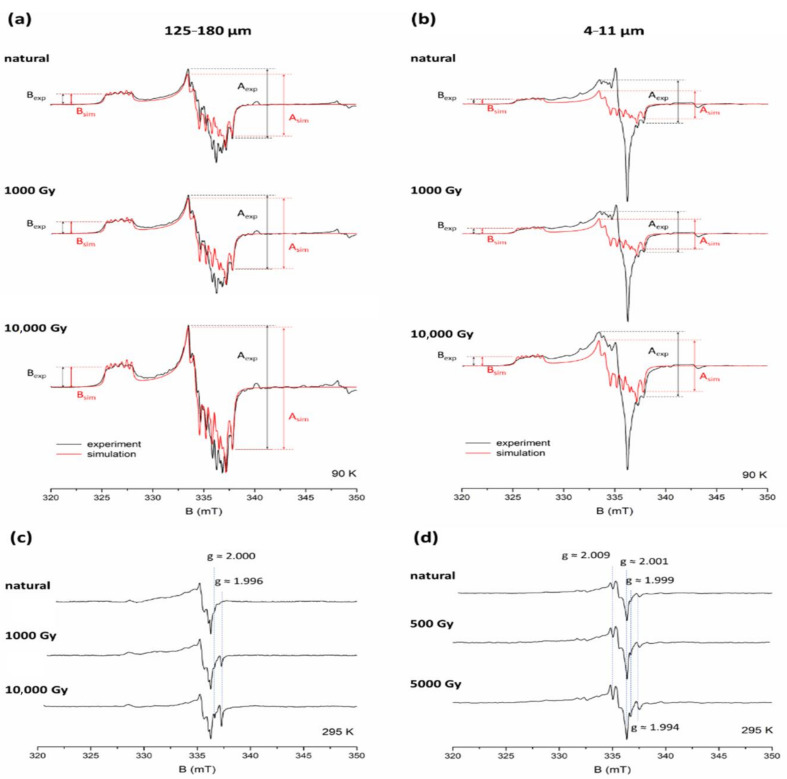
EPR spectra of the coarse (125–180 μm) and fine (4–11 μm) fraction of sample ROX 1.14, natural and additionally irradiated with 1000 and 10,000 Gy, recorded at 90 K ((**a**,**b**), respectively) and at room temperature ((**c**,**d**), respectively), and simulated spectra of Al-h signal (in red). Amplitudes A_exp_, A_sim_, B_exp_ and B_sim_ are marked with arrows. Major dose-dependent signals observed at room temperature are marked with blue dashed lines. The 90 K spectra for coarse grains are multiplied by a factor of 2.6.

Figure 2 shows a comparison of coarse (125–180 μm) and fine (4–11 μm) quartz EPR spectra for sample ROX 1.14 acquired at 90 K and at room temperature. Both fractions exhibit clear differences in the shape of the spectra. Experimental spectra of natural and additionally irradiated (with 1000 and 10,000 Gy) samples recorded at 90 K were overlaid with a simulated spectrum of Al-h (Figure 2a,b). The shape of the experimental spectra differs from the simulated one, which is caused by overlapping with signals assigned to the so-called peroxy species. The difference between Al-h simulation and experimental spectra recorded at 90 K is much more significant in the case of fine grains, as the peroxy signals in 4–11 μm quartz are much stronger than in the bigger fractions, which was previously reported by Timar-Gabor [22]. In the case of coarse grains, this difference is visible only in the centre of the spectra and remains more pronounced for the smaller doses of irradiation. 

The peroxy signals can be clearly registered at room temperature, when Al-h signal is not detectable (Figure 2c,d). The structure of the spectra is complex and consists of several overlapping signals. Their detailed characterisation and interpretation have been a subject of several studies (e.g., [29,30,31,32,33,34]) and is beyond the scope of this work. What is relevant for this study is whether the intensity of some of these signals depends on the dose of irradiation, an issue which, to our knowledge, has not been addressed in the literature. The only exception is a mention of a dose-dependent signal at g ≈ 2.011 detected by Timar-Gabor [22] in fine grains. The spectra of coarse-grained quartz shown in Figure 2c indicates that only two signals, at g ≈ 2.000 and g ≈ 1.996, increase with the applied dose, while the rest do not show any changes. The signal at g ≈ 2.000 can be ascribed to the E’ centre and the peak at g ≈ 1.996 to the Ge centre, namely, [GeO_4_/Li^+^]^0^ [5] (their EPR spectra can be found therein). The peroxy signals detected in fine grains (Figure 2d) are much stronger, and the presence of some additional peaks is visible. In addition to the E’ signal observed at g ≈ 1.999 and the Ge signal at g ≈ 1.994, at least two other signals, at g ≈ 2.009 and g ≈ 2.001, also show an increase with an increasing dose. The presence of these dose-dependent signals strongly influences the overall shape of the spectrum at high doses. The precise relationship between the intensity of these signals and the dose of laboratory irradiation cannot be determined at this point, as it requires separating them from the overlapping peaks, which is not possible without the aid of simulations and/or measurements at higher microwave frequencies.

As the peroxy signals in the 125–180 μm fraction of ROX 1.14 (and most of them in the case of the fine grains) are not dose-dependent, their contribution to the overall intensity of the signals registered in the considered range at low temperature decreases with the dose of radiation due to the increase in the Al-h signal. For coarse grains (Figure 2a), the experimental spectrum at high doses is very close in shape to the simulated one, while for fine grains (Figure 2b), the difference is still clearly visible.

When overlaying the experimental spectrum with a simulated one, we were faced with the issue of properly adjusting the amplitude of the latter. Since the central part of experimental spectra has proven to be distorted, to a varying degree, it should not be used as a reference point to adjust the simulated spectra. Therefore, a logical course of action was to choose peaks in the low-field part of the spectral range, specifically, the centre of the peak around g ≈ 2.0603 (see Figure 1), and match the amplitude of the simulated spectrum to the experimental spectrum each time, using this point as a reference.

The interference of peroxy signals may naturally cause problems for accurate measurements of Al-h signal intensity. A well-established method of measuring the intensity of the Al-h signal is based on the measurement of peak-to-peak amplitude between the top of the first peak of the central signal (g = 2.018) and the bottom of the last peak (g = 1.993) (see Figure 1) [27]. In Figure 2a,b we mark the amplitudes measured using this approach (denoted further as “A”), obtained from the experimental (A_exp_) and simulated (A_sim_) spectra of ROX 1.14 sample. As demonstrated for the additional dose of 10,000 Gy, both A_exp_ and A_sim_ give basically the same value (less than 2% difference) for coarse grains at high doses. However, due to the greater contribution of peroxy signals, A_exp_ amplitude at low doses is slightly overestimated compared to A_sim_ for the natural sample, giving about 13% and, for 1000 Gy, about 5% higher value compared to A_sim_. For fine grains, the overestimation of A_exp_ compared to A_sim_ is much more significant. For the natural sample of 4–11 μm ROX 1.14, it amounts to approximately 54%; for natural + 1000 Gy, about 38%; and for natural + 10,000 Gy, about 27%, as a result of the increasing contribution of dose-dependent Al-h signals and the decreasing contribution of mostly non-dose-dependent peroxy signals.

It is therefore clear that, although the approach based on measuring amplitude A works very well for samples of coarse-grained quartz which have accumulated a high dose of irradiation (e.g., very old samples and laboratory-irradiated samples), it can result in a significant overestimation in the case of fine-grained and young coarse-grained quartz, which can affect the slope of the dose response curve. A more reliable method for quantitatively describing the changes in Al-h concentration with the dose would be using the simulated signals and calculating the area under the curve with double integration. This value is directly proportional to spin concentration and will not be affected by any contributions from other paramagnetic species. Despite these advantages, this method is very time consuming, demands more signal processing and is not always accessible. However, as mentioned previously, adjusting the simulated spectrum to the experimental one requires a reference point (or, to be precise, a second reference point, the first being the baseline), which in this case, was chosen as the centre of the peak around g ≈ 2.0603 (see Figure 1). This provides the possibility of obtaining a reliable measurement of the amplitude simply by measuring the peak-to-baseline height of this peak of the experimental spectra, further referred to as “B”. The values of B_exp_ and B_sim_ (Figure 2) will therefore always be, by definition, equal to each other for every example of coarse and fine spectra. This approach allows for a much more accurate representation of Al-h signal intensity for fine grains, and may also improve the measurement of coarse grains, particularly for younger samples.

**Figure 3:**
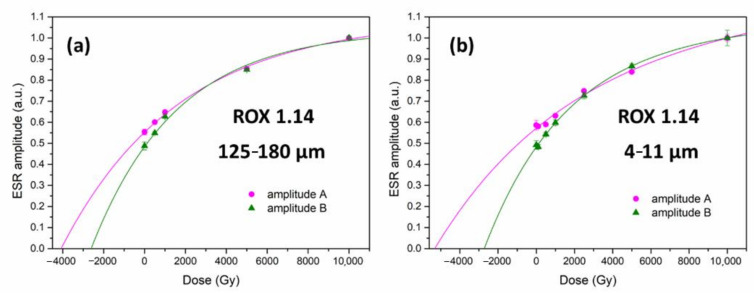
Dose response curves obtained for samples ROX 1.14 125–180 μm (**a**) and 4–11 μm (**b**) by measuring amplitudes A_exp_ and B_exp_. Data normalised by the maximum value. Negative dose values indicate the dose accumulated in the material prior to laboratory irradiation.

To investigate the effect of this overestimation on the shape and slope of the dose response curve (DRC), two sets of DRCs were constructed for sample ROX 1.14 (coarse and fine grains), using amplitudes A_exp_ (DRC A) and B_exp_ (DRC B) (Figure 3a,b). A sum of two exponential functions was used to fit the datapoints.

As expected from the comparison between simulated and experimental spectra, the lower dose part of DRC A for coarse grains (Figure 3a) bends upwards compared to DRC B due to the contribution from peroxy signals, while at higher doses, curves A and B overlap. As a result, the equivalent dose obtained from DRC A is overestimated. The divergence between DRC A and B is more pronounced in the case of quartz fraction 4–11 μm (Figure 3b). Because of the peroxy contribution, DRC A has a much smaller slope, which leads to a considerable overestimation of the equivalent dose obtained from this curve compared to curve B. It should be kept in mind that, while both these curves seem to almost overlap at high doses, the values of amplitude A are still over 25% overestimated compared to amplitude B at 10,000 Gy. Since the exact nature of the dose-dependency of the signals observed in the case of fine grains is not known, the relationship between A and B values for doses above 10,000 Gy cannot be predicted at this point, and may further affect the shape and slope of DRC A.

#### 3.1.2. Sample STY 1.10

**Figure 4:**
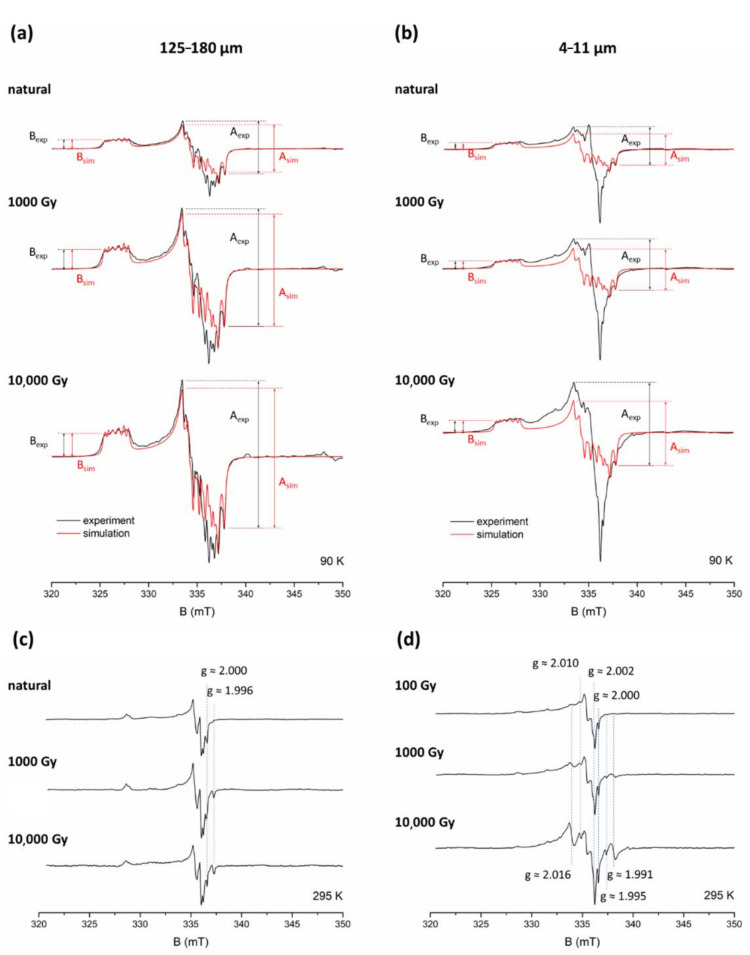
EPR spectra of the coarse (125–180 μm) and fine (4–11 μm) fraction of sample STY 1.10, natural and additionally irradiated with 1000 and 10,000 Gy, recorded at 90 K ((**a**,**b**), respectively) and at room temperature ((**c**,**d**), respectively), and simulated spectra of Al-h signal (in red). Amplitudes A_exp_, A_sim_, B_exp_ and B_sim_ are marked with arrows. Major dose-dependent signals observed at room temperature are marked with blue dashed lines. The 90 K spectra for coarse grains are multiplied by a factor of 5.9.

The second example of comparison between Al-h measurements for coarse and fine grain spectra is sample STY 1.10 (Figure 4). The spectra of the 125–180 μm fraction demonstrate a similar situation to ROX 1.14—the shape of experimental spectra differs slightly from the simulation for smaller doses, and this difference becomes less significant as the radiation dose increases (Figure 4a). The value of A_exp_ amplitude overestimates A_sim_ by about 13% for the natural signal, and about 5% for 1000 Gy and 10,000 Gy. For fine grains represented by a 4–11 μm fraction (Figure 4b), the spectra for the natural sample and the sample irradiated with 1000 Gy show differences between simulation and experiment analogous to the ones observed for sample ROX 1.14. Due to the contribution of peroxy signals, A_exp_ overestimates A_sim_ by about 24% for the natural sample and about 21% for 1000 Gy irradiated sample. However, for higher doses, the situation becomes even more complex, as the overestimation of A_exp_ compared to A_sim_ increases again, to about 30% for 10,000 Gy. The explanation for this fact comes from analysing the peroxy signals observed at room temperature (Figure 4c,d). While the spectra of coarse grains (Figure 4c), as in the case of sample ROX 1.14 (Figure 2c), do not show significant changes as the dose increases, with only the Ge signal at g ≈ 1.996 and E’ signal at g ≈ 2.000 being more prominent at high doses, the spectra of fine grains (Figure 4d) exhibit some additional signals, which increase their intensity with the laboratory dose. Most of them—the signal at g ≈ 2.002, 2.010, the Ge signal (g ≈ 1.995) and the E’ signal (g ≈ 2.000)—are also detected in sample ROX 1.14 (Figure 2d), but two other signals at g ≈ 1.991 and 2.016 are not. In particular, the signal at g ≈ 2.016 exhibits a considerable growth, and due to its position, which almost coincides with the top of the first peak of the central Al-h signal (g = 2.018) used for A_exp_ estimation, it strongly affects the outcome of this measurement for higher doses. As a result, A_exp_ amplitude obtained for fine grains provides unreliable measurements not only for lower doses, but also for higher doses, making it unsuitable for Al-h intensity determination. It is worth mentioning that, at first glance, the low-temperature spectrum of STY 1.10 irradiated with 10,000 Gy does not show clear signs of distortion around g = 2.018, as it still resembles the shape of the Al-h signal quite well, which can be very misleading, as it encourages attempts to measure A_exp_. It is only through analysing the room temperature measurements that the dose-dependent nature of the signal at g ≈ 2.016 can be revealed. In cases such as this, measuring A_exp_, although technically possible, results in unreliable data, leading to a distorted shape in the dose-response curve. Amplitude B, however, remains unaffected by the contribution of other signals, and therefore provides a reliable representation of Al-h signal intensity changes.

#### 3.1.3. Sample 2 MV 80

**Figure 5:**
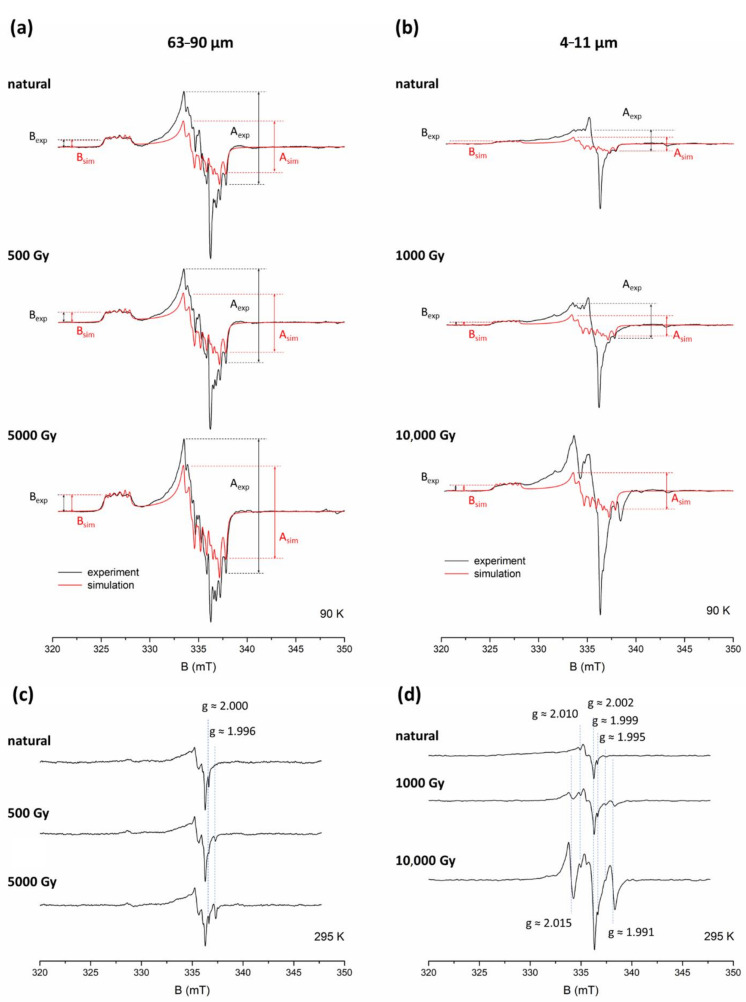
EPR spectra of the coarse (63–90 μm) and fine (4–11 μm) fraction of sample 2 MV 80, natural and additionally irradiated with 500 and 5000 Gy for coarse grains, and 1000 and 10,000 Gy for fine grains, recorded at 90 K ((**a**,**b**), respectively) and at room temperature ((**c**,**d**), respectively), and simulated spectra of Al-h signal (in red). Amplitudes A_exp_, A_sim_, B_exp_ and B_sim_ are marked with arrows. Major dose-dependent signals observed at room temperature are marked with blue dashed lines. The 90 K spectra for coarse grains are multiplied by a factor of 5.6.

The third example is based on measurements of sample 2 MV 80 (Figure 5). The spectra recorded at 90 K for coarse grains (Figure 5a), in this case represented by a 63–90 μm fraction, show a more significant distortion than in the case of samples ROX 1.14 and STY 1.14. This is most likely due to the smaller size of the coarse grains—63–90 μm instead of 125–180 μm—compared to the other two samples. As shown by Timar-Gabor [22], the intensity of peroxy signals decreases with increasing grain size. For the natural sample, A_exp_ overestimates A_sim_ value by as much as 82%, by 62% for 500 Gy and by 44% for 5000 Gy. A small part of this overestimation might be attributed to performing a baseline correction, as the original baselines displayed a steeper slope and more complex shape, but even then, the differences between A_exp_ and A_sim_ are still very considerable. Due to a limited availability of coarse material, fewer additional doses could be investigated; therefore, the overestimation present at 10,000 Gy could not be determined. The spectra recorded at room temperature resemble those obtained for samples ROX 1.14 and STY 1.10, with the same dose-dependent signals at g ≈ 2.000 and g ≈ 1.996, ascribed to the E’ and Ge centre, respectively, being visible.

As with samples ROX 1.14 and STY 1.10, for fine grains (Figure 5b), the contribution of the peroxy signals in the central part of the spectrum is clearly visible, leading to an overestimation of A_exp_ by 50% compared to A_sim_ for the natural sample and 64% for the 1000 Gy irradiated sample. At the higher doses, as shown for 10,000 Gy, the spectrum becomes very distorted, to the point that the measurement of A_exp_ is basically impossible, as it is clearly too affected by the overlapping signals. Measurements performed at room temperature (Figure 5d) show the same dose-dependent signals, as in the case of the samples ROX.1.14 and STY.10—at g ≈ 2.010, 2.002, and at g ≈ 1.999 (E’ centre) and 1.995 (Ge centre), as well as very strong dose-dependent signals at g ≈ 2.015 and g ≈ 1.991, also observed in sample STY 1.10, in addition to the non-dose-dependent signals, also visible in the coarse grains. In the case of samples like 2 MV 80, with very strong dose-dependent signals overlapping with the Al-h signal, the measurement of amplitude B is not only more reliable, but also appears to be the only viable option for obtaining Al-h amplitude without the use of simulations. It should be noted that the presence of dose-dependent signals will also cause problems when attempting to remove the peroxy signals by subtracting the spectra recorded after heating (as performed by Richer and Tsukamoto [36]), since the peroxy spectrum will look different for every dose, and the heating will likely affect the ratio of dose-dependent and non-dose-dependent peroxy signals. These arguments further support using amplitude B for Al-h intensity determination for both fine and coarse grains.

The comparison between the results obtained for the three presented examples by measuring the amplitude A and B shows the advantage of using amplitude B for Al-h intensity estimation. Contrary to amplitude A, it is not affected by the peroxy signals present in the centre of the analysed range, and it can therefore provide more accurate results, or, in fact, any results in cases where a spectrum is too distorted to allow for an estimation of amplitude A. Measurements of amplitude B were used in the second part of this study to compare the response of coarse and fine grains of quartz to laboratory irradiation.

### 3.2. Comparison of DRCs of Coarse and Fine Grains

**Figure 6:**
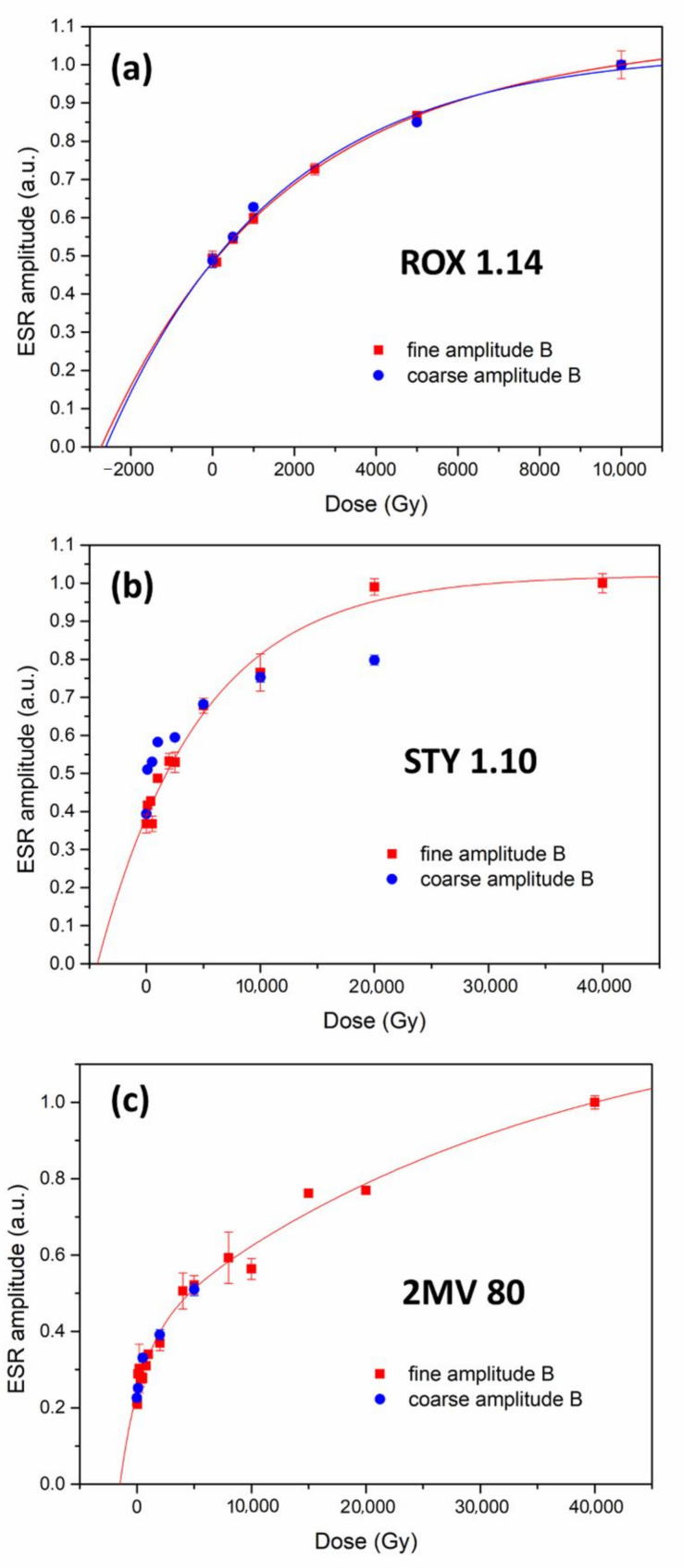
Dose response curves for the amplitude B_exp_ obtained for fine (4–11 μm) and coarse (125–180 μm) grains of samples ROX 1.14 (**a**), STY 1.10 (**b**), and fine (4–11 μm) and coarse (63–90 μm) grains of sample 2 MV 80 (**c**). Data were normalised to the maximum datapoint (**a**) or maximum datapoint for fine grains and overlaid on the curve for coarse grains (**b**,**c**). Negative dose values indicate the dose accumulated in the material prior to laboratory irradiation.

Dose response curves obtained using amplitude B_exp_ were used for comparing the response of coarse and fine grains of quartz to laboratory irradiation (Figure 6). The behaviour of the Al-h signal was investigated up to 10,000 Gy on top of the natural dose for sample ROX 1.14, and up to 40,000 Gy for samples STY 1.10 and 2 MV 80. Due to the limited availability of the material, fewer aliquots were obtained for the coarse fraction than the fine fraction. No correction for the residual dose was applied.

A note of caution regarding the fitting is necessary before proceeding to describe these results. A sum of two saturating exponential functions was used to fit the data. This choice was dictated by the results obtained in a recent study by Benzid and Timar-Gabor [43], where a phenomenological model of Al-h formation upon room temperature irradiation was proposed. In this model, the Al-h centre is considered to be formed upon laboratory irradiation by two processes: (i) directly by transforming [AlO_4_/M^+^]^0^ into Al-h, and (ii) indirectly by transforming [AlO_4_/M^+^]^0^ into [AlO_4_/H^+^]^0^, and then [AlO_4_/H^+^]^0^ into Al-h. By assuming that the dissociation rates of these centres are proportional to their concentrations, the model shows that the increase in the Al-h EPR signal with increasing dose can be well described by a sum of two exponential functions. Benzid and Timar-Gabor, however, acknowledge the dangers of interpreting the parameters derived through fitting with multiple exponentials, stating that, for quantitative assumptions using the derived parameters to be made, the DRC needs to be raised until it reaches full saturation; otherwise, the parameters depend on the maximum given dose, as was shown previously by Timar-Gabor et al. [21] for DRCs obtained for OSL signals fitted with a sum of two saturating exponentials. As is clear from Figure 6, this is not the case for DRCs constructed in the current study; therefore, we refrain from deriving any conclusions based on parameters obtained from the fittings. As such, the fitted curves presented in Figure 6 should be regarded primarily as a visual aid in comparing the response to laboratory irradiation. Additionally, due to a smaller number of datapoints for coarse-grained samples STY 1.10 and 2 MV 80 and their noticeable scatter, a fitting was not performed.

Proceeding to the comparison of fine and coarse quartz DRCs, it is immediately apparent from Figure 6a that both the 4–11 μm and 125–180 μm fractions of the ROX 1.14 sample show almost identical DRC shape. While the number of datapoints is limited, it can be assumed that the effect of increasing the laboratory dose on the Al-h signal, even if not identical, is remarkably similar in fine and coarse grains. As mentioned before, the data obtained for the coarse fraction of samples STY 1.10 (Figure 6b) 2 MV 80 (Figure 6c) did not allow for a satisfactory fitting, as the shape of the fitted curves would be largely affected by an arbitrary choice of parameters. Instead, the datapoints were overlaid on the DRCs obtained for fine grains. Some differences can be observed for the sample STY 1.10 (Figure 6b), namely, the datapoints obtained for coarse (125–180 μm) grains seem to indicate a faster saturation of the DRC. However, the shape of the curves is likely affected by a noticeable scatter of the datapoints and the absence of data for coarse grains above 20,000 Gy, so the divergence observed in Figure 6b may very well be exaggerated. In the case of sample 2 MV 570 (Figure 6c), as far as the doses up to 5000 Gy are concerned, the data for coarse (63–90 um) and fine grains is in very good agreement, suggesting that, in this range, there are no significant differences in the behaviour of the Al-h signal in coarse and fine grains for this sample. Despite the aforementioned issues with the fitting, simply by visually following the datapoints, it can be observed that, in all three cases, the intercept of the DRCs with the x axis seem to be the same for both fractions.

It can therefore be stated that no significant divergence in the behaviour of the Al-h signal with the increasing radiation dose in the investigated range can be observed between the fine (4–11 μm) and coarse (63–90 μm and 125–180 μm) grains of quartz studied in this work. A logical conclusion is to assume that Al-h centre formation in fine and coarse grains due to artificial radiation is governed by the same processes. To our knowledge, the influence of grain size on the formation of the Al-h centre has not been discussed in the literature. The phenomenological model of Al-h formation upon laboratory irradiation at room temperature proposed by Benzid and Timar-Gabor [43] does not suggest that this process would be significantly different for coarse and fine grains. As Al-h centres are extrinsic, impurity-related defects, it is to be expected that they would have a relatively homogeneous distribution in the volume of a sedimentary quartz grain. Indeed, Timar-Gabor [22] report that no significant effect could be observed when measuring Al-h signals as a function of etching time. More experimental and theoretical studies are certainly needed to further examine the mechanism of Al-h centre formation; however, our results show that, in the first approximation, the response of both fine- and coarse-grained quartz to artificial irradiation is remarkably similar.

Should coarse and fine grains of quartz therefore be provided with the same equivalent dose when dated using the Al-h centre? The answer to this question requires a separate consideration and cannot be answered at this point. It is generally accepted that sunlight exposure does not completely bleach the EPR intensity of Al-h, and the signal is reset only to a non-bleachable residual level (e.g., [6,7,44,45,46,47,48]). Our study focuses on unbleached samples, which have a residual signal composed of bleachable and unbleachable components. These components can be of different magnitudes for coarse and fine grains and should be determined separately for every fraction, which is beyond the scope of this work. To our knowledge, the only study showing the effects of grain size on the results of the EPR dating of quartz was conducted by Liu et al. [49] for the Ti-Li centres of fluvial and lacustrine sediments. They assumed complete bleaching and reported that, for grain sizes above 100 μm, the equivalent dose decreased with the increase in grain size. However, for the smallest fraction (50–100 μm, which, in our study, would still be considered coarse), the equivalent dose was smaller than for the larger fraction. They also showed that the beta irradiation dose rate of grains with different sizes accounts for only about 6% of the total deviation of dating results, making if far less significant than the effect of grain size on EPR sensitivity. No similar studies have been conducted on the Al-h centre of sedimentary quartz. It should be mentioned that the effect that the size of grains has on the obtained equivalent dose was investigated for E’ and the Al-h centre in quartz from fault gouge (e.g., [50,51]), but due to the different mechanism involved in resetting the signal (mechanical deformation and high temperature), these results cannot be of use for other types of environments. The effects of natural irradiation and light exposure on fine- and coarse-grained quartz should certainly be investigated in order to reach conclusions regarding the equivalent dose estimation.

## 4. Conclusions

We examined the Al-h and peroxy EPR spectra of fine (4–11 μm) and coarse (63–90, 125–180 μm) sedimentary quartz separates extracted from three well-characterised samples collected from thoroughly investigated sites (Roxolany, Stayky and Mircea Vodă). Based on the data presented in this work, as well as in the study conducted by Timar-Gabor [22], it is clear that Al-h measurements of fine grains are affected by the presence of peroxy signals to a much greater extent than coarse grains. However, the degree to which this affects the standard amplitude measurement following the approach of Toyoda and Falguères [27] seems to be sample-dependent. It ranges from causing an overestimation, which is much stronger for smaller doses (sample ROX 1.14), to a complete distortion of the spectra at high doses (sample 2 MV 80) due to the presence of dose-dependent peroxy signals in fine grains. For a proper understanding of the observed differences, a much larger set of samples would certainly have to be analysed, which is not an easy task, since it requires a large amount of material from different sites divided into fractions. 

The new approach to measuring Al-h signal amplitude proposed in this study, focusing on the peak-to-baseline amplitude of the part of the signal at g ≈ 2.0603, has the potential to provide more accurate results. This region of the spectrum is not affected by strong peroxy signals overlapping the central part of the Al-h signal and causing the overestimation. While using the strongest absorption line, as in the standard approach, increases the signal-to-noise ratio, which leads to greater precision in the dating result, our study shows that this precision comes at the cost of accuracy. In other words, while the errors associated with the standard approach may be smaller, the dates themselves may not reflect the true age of the material. We believe that more accurate results, even if less precise, are of much greater value to the dating community and the researchers using the reported values in their studies. It should be mentioned that, while very useful for samples with strong Al-h signals, as the ones investigated here, the new approach might not be applicable to very weak signals. This part of the Al-h spectrum is considerably less intense than the central signal typically used for measuring amplitude, and in the case of some samples, it may simply be undetectable. Additionally, more studies are needed on the individual signals composing the peroxy spectra in order to rule out the possibility of some weaker lower-field peaks being present around g ≈ 2.0603, which could affect the amplitude measurement following this new approach.

We compared the response of the Al-h signal to laboratory irradiation displayed by the fine- and coarse-grained fractions, which has not been previously shown in the literature. The shapes of dose response curves constructed for coarse and fine grains using the new approach show a considerable similarity, which suggests that Al-h centre formation in fine and coarse grains upon artificial radiation follows the same pattern. These observations have significant implications for the dating community and will hopefully inspire more research, experimental and theoretical, allowing for a thorough comparison of dating results obtained for different fractions of sedimentary quartz, which in turn will deepen our understanding of the underlying processes and increase the accuracy of EPR dating.

It should be stressed that the behaviour of the Al-h signal in coarse and fine grains upon laboratory irradiation might differ from behaviour observed in nature. Depending on the grain size, the amount of alpha and beta radiation penetrating the grain will be different, influencing the formation of defects. Understanding the processes induced in fine and coarse grains by gamma radiation in a controlled laboratory environment is the first step towards the development of a comprehensive model. The effect of grain size on the formation and bleachability of Al-h centres under natural conditions needs to be thoroughly studied before any conclusions are drawn regarding the overall result of EPR dating using different fractions. We hope that our work will stimulate such studies.

## Figures and Tables

**Table 1:** Spin Hamiltonian parameters used for simulating [AlO_4_]^0^ spectrum. A—hyperfine splitting, Q—quadrupole splitting. S = 1/2, ^27^Al (I = 5/2), Lorentzian peak-to-peak linewidth 0.185 mT.

Parameter		x	y	z
g-Tensor		2.0603	2.0083	2.0021
A	(MHz)	14	17	18.2
	(mT)	0.499	0.606	0.649
Q	(MHz)	−0.62	−0.43	1.05
	(mT)	−0.022	−0.015	0.037

## Data Availability

The data analysed in this study are available from the corresponding authors upon reasonable request.
